# Newly diagnosed type 1 diabetes mellitus in a human immunodeficiency virus-infected patient with antiretroviral therapy-induced immune reconstitution inflammatory syndrome: a case report

**DOI:** 10.1186/s12879-023-08605-1

**Published:** 2023-09-20

**Authors:** Min-ChunYeh, Han-Chuan Chuang, Shuen-Fu Weng, Chung-Huei Hsu, Chen-Ling Huang, Yu-Pei Lin, Yan-Yu Lin, Yu-Shan Hsieh

**Affiliations:** 1 Division of Endocrinology and Metabolism, Department of Internal Medicine, Taipei Medical University Hospital, Taipei City, Taiwan; 2 Division of Infection Diseases, Department of Internal Medicine, Taipei Medical University Hospital, Taipei City, Taiwan; 3 Division of Endocrinology and Metabolism, Department of Internal Medicine, School of Medicine, College of Medicine, Taipei Medical University, Taipei City, Taiwan; 4 School of Nursing, National Taipei University of Nursing and Health Sciences, No. 365, Mingde Rd., Beitou Dist, Taipei City, 112303 Taiwan; 5 Department of Research, Taipei Medical University Hospital, Taipei City, 11031 Taiwan

**Keywords:** Type 1 diabetes mellitus, Graves’ disease, Immune reconstitution inflammatory syndrome, Human immunodeficiency virus, Antiretroviral therapy

## Abstract

**Background:**

Diabetes that develops in human immunodeficiency virus (HIV)-infected patients who receive antiretroviral therapy (ART) is usually type 2 diabetes mellitus (T2DM); however, autoimmune diabetes, such as type 1 diabetes mellitus (T1DM) can also develop in this population. After treatment with ART, patients might experience clinical deterioration following an increase in the CD4 cell count, which is termed immune reconstitution inflammatory syndrome (IRIS). Here, we describe an HIV-infected patient on ART who developed T1DMat due to IRIS, highlighting the clinical complexity in diagnosis and treatment.

**Case presentation:**

A 36-year-old man infected with HIV had a nadir CD4 cell count of 15.53/μL before medication, which increased to 429.09/μL after 9 months of regular ART. The fasting serum glucose at 9 months was between 96 mg/dL and 117 mg/dL. After 11 months of ART, the patient was admitted to hospital for diabetic ketoacidosis (DKA) and Graves’ disease (GD). Noninsulin antidiabetics (NIADs) were prescribed following the resolution of DKA. However, poor glycemic control was noted despite well-titrated NIADs. Further investigation demonstrated poor pancreatic beta cell function and elevated anti-glutamic acid decarboxylase (anti-GAD) and anti-tyrosine phosphatase-like insulinoma antigen 2 (anti-IA2) titers. According to the results, he was diagnosed with T1DM and received multiple daily injections(MDI) of insulin. The regimen of MDI was insulin degludec as basal insulin and insulin aspart as prandial insulin. After MDI therapy, his glycemic control was improved.

**Conclusion:**

In this case, T1DM was ascribed to IRIS. Although this phenomenon has been demonstrated in previous case reports, further study is necessary to realize the mechanism of this association. Therefore, we emphasize that when HIV-infected patients on ART experience an unstable blood glucose level and abnormal thyroid function, physicians should consider T1DM and GD associated with ART-induced IRIS to reduce the subsequent complications and more serious endocrine dysfunction.

## Background

Human immunodeficiency virus (HIV) affected approximately 0.5% of the global population at the end of 2021, with 650,000 deaths attributable to HIV-related illness. The induction of endocrine dysfunction by HIV itself, related opportunistic infections, immune activation, and antiretroviral therapy (ART) has been well recognized [1].

Autoimmune diabetes has also been reported in HIV-infected patients on ART, with approximately 2–14% of HIV-infected patients believed to have diabetes [2]. Autoimmune diabetes, such as type 1 diabetes mellitus (T1DM), can also be induced by HIV infection but is less commonly reported. In Taiwan, the incidence of T1DM is 2.23 per 100,000 person-years after adjusted for age [3]. Although the exact mechanism between diabetes and HIV infection is unclear, immune reconstitution is thought to play a role [4].

ART-like protease and nucleoside reverse transcriptase inhibitors can induce insulin resistance and represent risk factors. ART has been demonstrated to dramatically alter the course of HIV infection by decreasing the occurrence of opportunistic infections and mortality. However, after treatment with ART, patients might show clinical deterioration following an increase in CD4 cell count or a decrease in HIV viral loading, resulting in an immune response and severe inflammation, which is termed immune reconstitution inflammatory syndrome (IRIS) [5].

IRIS is a state of exaggerated inflammation that usually occurs in patients with HIV/AIDS initiating ART, largely owing to the restoration of immune function, including CD4-positive T cells [6]. The target antigens not only include pathogenic microorganisms but also self-antigens due to the development of autoimmune disease. Chen, Fabian et al. described 17 HIV-infected patients who developed autoimmune thyroid disease following 17 months of ART [7]. To date, some studies have reported that up to 11.1–22.9% of patients with HIV who are receiving ART also have IRIS [8]. Despite some reports of autoimmune diabetes in HIV-infected patients, the possible induced complication of IRIS has not yet been clarified.

Here, we describe the case of an HIV-infected patient on ART who developed T1DM due to IRIS, highlighting the clinical complexity in diagnosis and treatment.

## Case presentation

A 36-year-old man had presented with acquired immunodeficiency syndrome (AIDS) for 5 years with poor compliance to ART. He first presented to our Infection Clinic on September 17, 2018 for abdominal pain and body weight loss (from 76 kg to 72 kg, BMI: 23.2 kg/m^2^), at which time he had a low CD4 cell count (14.07/μL) and HIV viral load (99,801 copies/mL). He was also diagnosed with amebic infection, syphilis, and hepatitis B. Following diagnosis, co-formulated elvitegravir, cobicistat, emtricitabine, and tenofovir alafenamide (E/C/F/TAF) were used as ART. After 3 months of E/C/F/TAF, the CD4 cell count was increased to 74.53/μL and the HIV viral load was < 20 copies/mL. During the 6-month treatment period, his body weight increased from 72 to 85 kg, his BMI was 27.4 kg/m^2^, his fasting glucose was between 90 mg/dL and 111 mg/dL, and his Hemoglobin A1C (HbA1C) level was 5.2%. However, after 6 months of E/C/F/TAF, he was lost to follow-up at the Infectious Disease Clinic for 1 year. During this period, he had not taken any ART.

On March 25, 2020, he presented to the Infectious Disease Clinic for a skin rash over the trunk and limbs for 1 week. His CD4 cell count was 15.53/μL and his HIV viral load was 26,021 copies/mL. Additionally, his body weight had reduced from 85 kg to 71 kg during the year he was lost to follow-up. E/C/F/TAF was replaced with co-formulated bictegravir (BIC), emtricitabine (FTC), and tenofovir alafenamide (BIC/FTC/TAF) as ART. After 9 months of BIC/FTC/TAF, his CD4 cell count was increased to 429.09/μL and his HIV viral load was < 20 copies/mL, with good compliance during the treatment course. During this 9-month period, his body weight increased from 71 to 78 kg, his BMI was 25.2 kg/m^2^, and his fasting serum glucose was between 96 mg/dL and 117 mg/dL. On February 3, 2021, he presented to emergency room for abdominal cramping pain, nausea, and vomiting for 2 days. Exophthalmos and grade 1 A goiter were noted. No abdominal tenderness or rebounding tenderness was noted. His heart rate was between 152 and 161 bpm, and electrocardiogram (EKG) revealed paroxysmal supraventricular tachycardia (PSVT). Laboratory test showed high serum random glucose (598 mg/dL), metabolic acidosis (arterial blood gas pH: 7.137, HCO_3_: 10.0 mmol/L, PCO_2_: 30.2 mmHg), high serum beta hydroxybutyrate (8.10 mmol/L, normal range:0.03–0.30 mmol/L), and glycated hemoglobin (HbA1C) of 8.5%. Thyroid function test showed primary hyperthyroidism (TSH < 0.01 μIU/mL, normal range 0.27–0.42 μIU/mL; free thyroxine (FT4) > 7.77 ng/dL, normal range 0.93–1.70 ng/dL) and positive TRAb titer (87.65%). Diabetic ketoacidosis (DKA) due to type 2 diabetes mellitus (T2DM) and Graves’ disease (GD) were suspected. As a result, he received a regular human insulin pump for DKA at the intensive care unit. After DKA was relieved, he was shifted to noninsulin antidiabetics (NIADs; metformin [500 mg] 1 tab TID, linagliptin [5 mg] 1 tab QD, and glimepiride [2 mg] 1 tab QD) at the general ward, where methimazole was used for GD. He was discharged with the NIAD regimen mentioned above and methimazole on February 8, 2021.

**Fig. 1:**
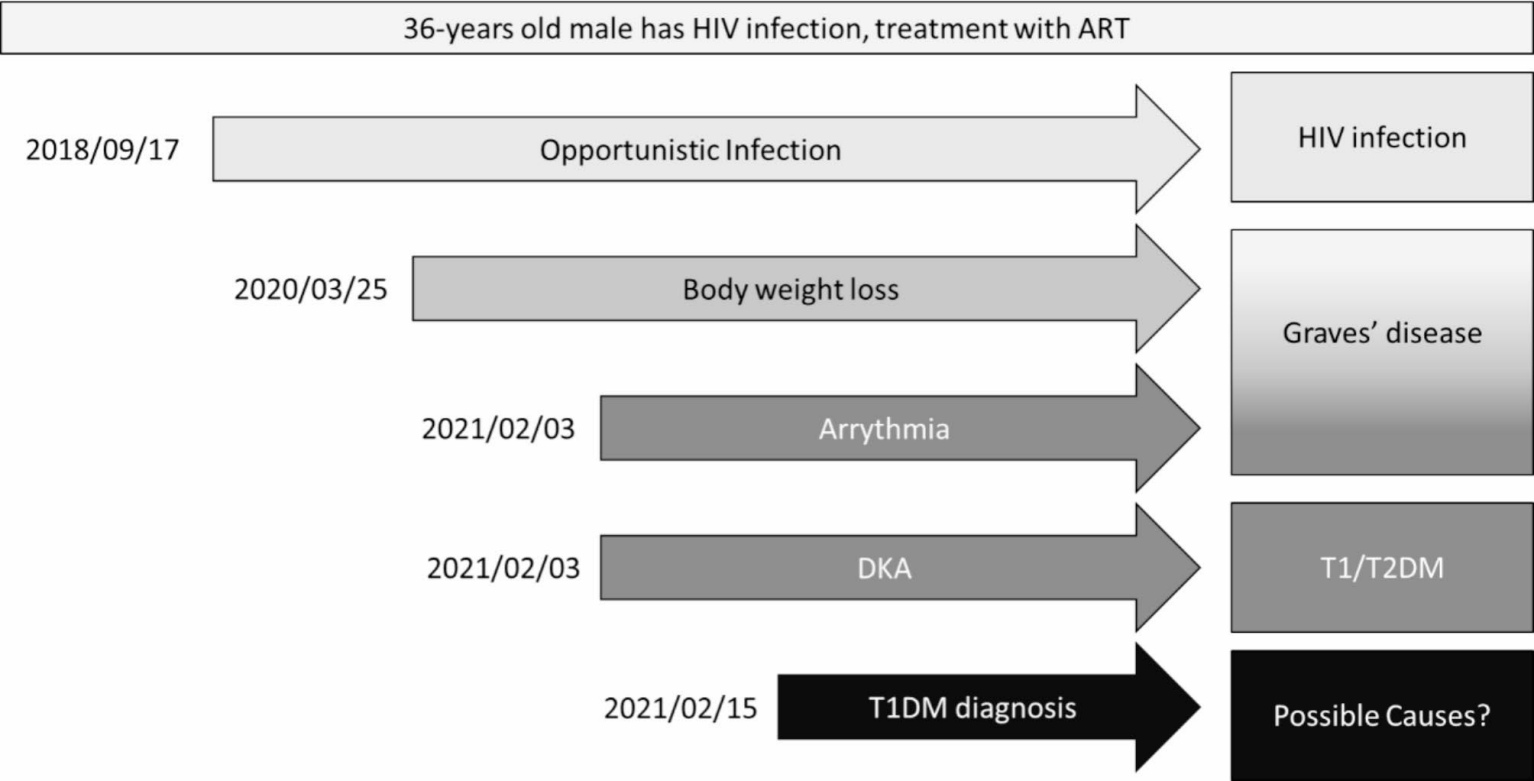
Course of progression of the current case. T1/T2DM, Type 1 or 2 diabetes mellitus; DKA, diabetic ketoacidosis

At the Endocrine and Metabolic Disease Clinic on February, 15, 2021, the patient was noted to have high postprandial serum glucose (360 mg/dL). A glucagon stimulation C-peptide test was performed, and the result showed poor pancreatic beta cell function (before 1 mg glucagon: 0.48 ng/mL, 6 min after 1 mg glucagon: 0.47 ng/mL). Consequently, his anti-glutamic acid decarboxylase (anti-GAD) and anti-tyrosine phosphatase-like insulinoma antigen 2 (anti-IA2) antibodies were checked; both were positive (anti-GAD > 2000 [+], Anti-IA2 47.8 [+]) and T1DM was diagnosed as a result. The NIAD regimen was replaced with an insulin basal-bolus regimen (insulin degludec 10U QD with insulin aspart 4U TIDAC initially). His compliance to insulin replacement therapy was inconsistent. The last recorded HbA1C was 11.4% and fasting glucose was 268 mg/dL under insulin degludec 36U QD and insulin aspart 14U TIDAC. The course of progression this case is presented in Fig. 1.

## Discussion and conclusion

Here, we reported a case of an individual with AIDS who developed DKA after 11 months of ART treatment. It was important to determine the cause of DKA, the type of diabetes mellitus that the patient acquired, and why the patient had acquired diabetes mellitus.

Nolan et al. described three patients who developed DKA within 3 weeks to 4 months after transition to BIC/FTC/TAF. None of the three patients had previously received medications for diabetes, with only modest elevations in HbA1c values before starting BIC/FTC/TAF(5.6–6.6%). The authors suggested that beta-cell dysfunction and/or insulin resistance independent of weight gain due to BIC/FTC/TAF may have accounted for the rapid acceleration of hyperglycemia [9]. However, in our patient, the use of BIC/FTC/TAF is less likely to explain the development of DKA due to the longer period between the initiation of medication and the development of DKA.

Autoimmune diabetes is subclassified as adult onset T1DM, latent autoimmune diabetes in the adult (LADA), and T2DM. In Taiwan, most new-diagnosed cases of diabetes mellitus are T2DM, while T1DM is considerably less common (0.24% vs. 99.76% annual incidence in 2019). Among HIV-infected patients, the reported prevalence of diabetes mellitus is between 2% and 14% [1, 2]. Insulin resistance, rather than insulin deficiency, is the leading mechanism of diabetes in HIV-infected patients [10].

Takarabe et al. described three Japanese patients diagnosed with autoimmune diabetes after ART initiation, all of whom initially took NIAD or no antihyperglycemic medication [11]. These three patients were a 30-year-old man, a 31-year-old man and a 68-year-old woman. None of the three patients had a positive GAD-Ab test before ART initiation, at which point, their nadir CD4 cell counts were < 20 cells/L. The GAD-Ab tests became positive from 6 to 38 months after initiation of ART, which was accompanied by a dramatic increase in CD4 cell count. Simultaneously, HIV-infected patients on ART with higher CD4 cell counts were assosciated with beta cell dysfunction compared to either HIV negative patient or HIV-infected patients without ART after adjusting for effects of age, race, sex, body mass index, and smoking status [12]. Hence, the development of autoimmune diabetes in HIV-infected patients after immune restoration should be considered [11].

LADA is a slow-progressing type of autoimmune diabetes. As with T1DM, LADA may occur due to pancreatic beta cell dysfunction resulting in inadequate production of insulin [13]. Autoimmune antibodies can slowly destroy the pancreatic beta cells, with both LADA and T1DM reported to share characteristics of immunological dysfunction, such as pancreatic β cell-specific autoantibodies and alterations in the immunophenotype of immune cells [14].

Lane et al. reported the case of an African American HIV-infected patient who was diagnosed with T2DM less than 10 years after receiving ART [4]. NIAD was given initially and insulin replacement therapy was introduced 13 years after the initial T2DM diagnosis. Eighteen years after his initial T2DM diagnosis, he was referred to the Diabetes Clinic for further survey of poor glycemic control despite good compliance to insulin glargine and saxagliptin and close monitoring of his diet and activity. LADA was diagnosed after a positive GAD-ab test. The patient’s nadir CD4 count was 2 cells/μL which increased to 772 cells/μL when autoimmune diabetes was diagnosed. Immune reconstitution was considered as the etiology [4]. Moreover, Sims et al. demonstrated that while HIV-infected patients not receiving ART do not have worsening beta cell function or glucose homeostasis, immune reconstitution with ART may be associated with worsened beta cell function [12]. The level of beta-cell dysfunction in patients with LADA has been reported to be intermediate between those with T1DM and T2DM. Although patients usually respond initially to NIAD, the response declines with the deterioration in β-cell function [15].

Our patient had never been diagnosed with diabetes before he started to receive BIC/FTC/TAF for AIDS. He was initially treated with NIAD under the impression of T2DM but the treatment response scarcely. According to a previous study, LADA is diagnosed in antibody-positive patients ≥ 30 years of age who require insulin more than 6 months after diagnosis - a distinguishing feature from adult-onset T1DM [16, 17]. Therefore, LADA was not considered in our patient because insulin replacement was initiated within 6 months of diagnosis.

**Fig. 2:**
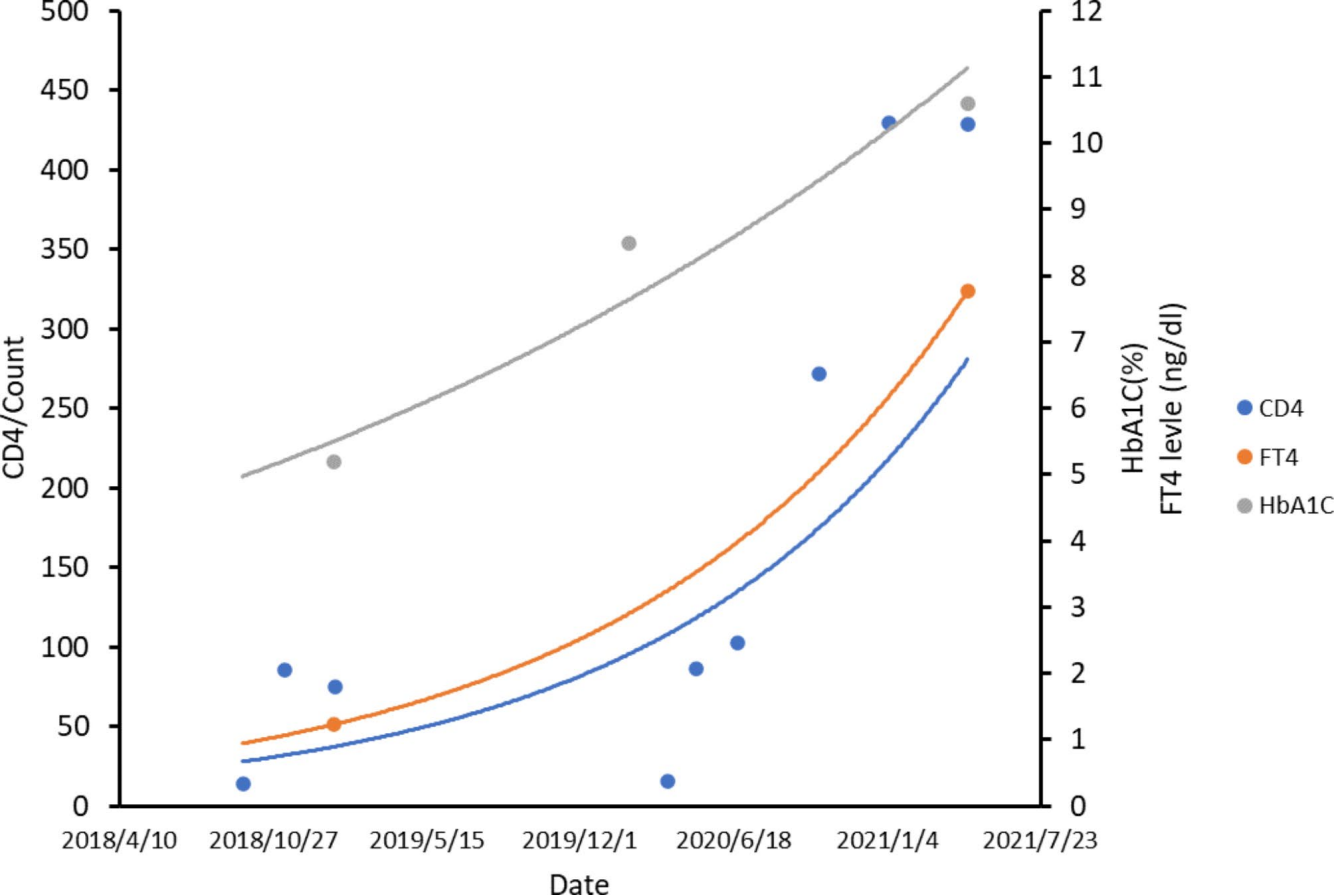
Relationship between the changes in CD4 count, FT4 level, and HbA1C levels. The curves were calculated by curvilinear regression. FT4, free thyroxine; HbA1C, glycated hemoglobin

In our patient, the laboratory results of the glucagon stimulation C-peptide test suggested poor beta cell function, and T1DM was diagnosed on the basis of positive anti-GAD and anti-IA2 antibodies during the period when the CD4 cell count was toward the normal range and the HIV viral load was suppressed. The relationship between the changes in CD4 count, FT4 level, and HbA1C level in this case are shown in Fig. 2. After HIV treatment, the levels of FT4 and HbA1C started to increase with the increasing CD4 count.

**Fig. 3:**
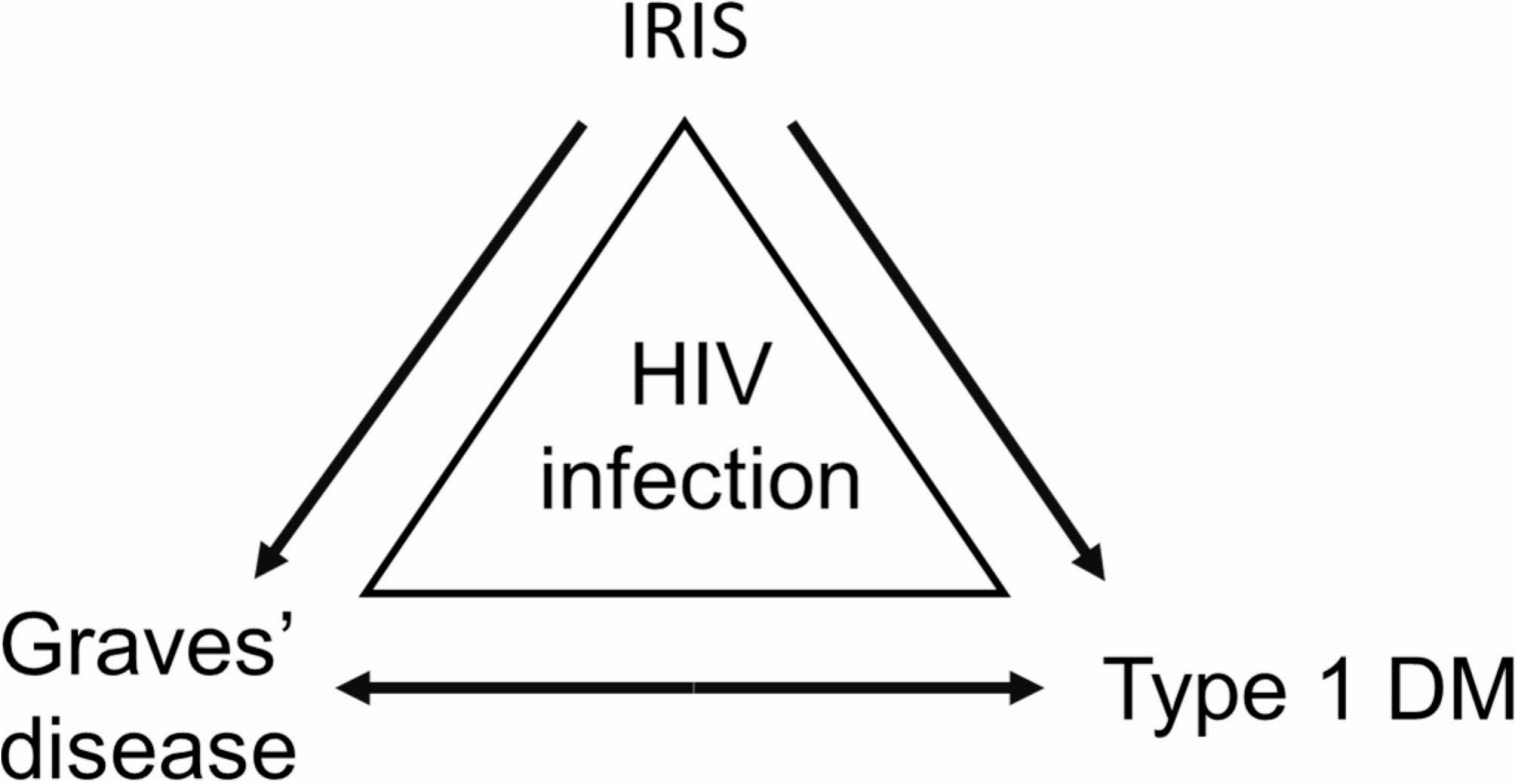
Proposed relationship between IRIS, T1DM, and GD. IRIS, immune reconstitution inflammatory syndrome; DM, diabetes mellitus; HIV, human immunodeficiency virus

In the current case, immune reconstitution may explain the development of T1DM. IRIS occurs in approximately 16.1% (range: 11.1–22.9%) of individuals with HIV who are on ART [8]. We hypothesize that the relationship between IRIS, T1DM, and GD can be explained as shown in Fig. 3. IRIS is an exaggerated immune response that destroys organs, with severe and irreversible consequences. The presentation of IRIS could be a new event or an exacerbation of infectious or non-infectious (e.g., autoimmune) disease, with risk factors including disseminated opportunistic infection and CD4 count < 50 cells/μL at the time of ART initiation [8, 18]. Regarding treatment for IRIS, in addition to immunosuppressors (e.g., corticosteroids), novel immune-modulators (e.g., molecular-targeted agents and checkpoint inhibitors) that target immune functions could also be considered [19].

In addition, thyroid dysfunction also can occur post-ART, among which, GD is the most common thyroid manifestation and may have a delayed presentation [20]. According to a previous study, GD occurred in more than 70 patients infected with HIV who received ART [20]. The onset of GD following ART has been described as 12–36 months following treatment, with a median onset of 21 months, which is consistent with the presentation of the current case [21]. However, to date, the pathogenesis of GD following ART is not fully understood. One possible mechanism is the loss of peripheral T cell tolerance, ultimately impacting tolerance to thyroid tissue [4, 22]. Although IRIS predominantly manifests as severe infection, it may also present as autoimmune diseases such as T1DM and GD.

We emphasize that ART may result in unstable blood glucose, abnormal thyroid function, and even the occurrence of DKA or thyroid storm. Clinical physicians should be aware of the association between ART-induced IRIS and T1DM and GD, which will assist with reducing the subsequent complications and occurrence of more serious endocrine dysfunction. Further study is necessary to realize the mechanism of beta cell dysfunction in such patients.

## Data Availability

The data used to support the findings of this study are included within the article.

## Abbreviations

ART: Antiretroviral therapy
GD: Graves’ disease
HIV: Human immunodeficiency virus
IRIS: Immune reconstitution inflammatory syndrome
NIAD: Noninsulin antidiabetic
T1DM: Type 1 diabetes mellitus.
